# In-Wall Imaging for the Reconstruction of Obstacles by Reverse Time Migration

**DOI:** 10.3390/s23094456

**Published:** 2023-05-03

**Authors:** M. Lütfi Yarar, Ali Yapar

**Affiliations:** Department of Electronics and Communication Engineering, Istanbul Technical University, Istanbul 34467, Turkey

**Keywords:** in-wall imaging, reverse time migration method, Monte Carlo simulation, quantitative performance evaluation, single frequency reconstruction, microwave imaging

## Abstract

In this paper, the reverse time migration (RTM) method is applied to the single-frequency reconstruction of embedded obstacles in a wall to perform an introductory study for in-wall imaging. The aim is to determine the geometrical properties of an object embedded in a wall by the use of an information function provided via the RTM method. The method is based on the computation of that information function separately at each point on a reconstruction domain. It is defined as the correlation levels between the incident fields emitted from sources and the back-propagation of the scattered field. The problem is taken from a broader perspective in order to show and confirm the effectiveness of the method. For this purpose, numerical experiments within a fundamental scenario are determined in a particular order to perform an essential Monte Carlo simulation. The paper uses a comparative study to make an objective evaluation of the achievement level of the method in in-wall imaging. The results reveal that the method is at the applicable level of achievement.

## 1. Introduction

The reverse time migration (RTM) method based on reverse time extrapolation is proposed in [1]. Besides many other migration algorithms [2], it is proven to be of high accuracy in the reconstruction of complex structures [3,4,5]. Among direct methods including [6,7,8,9,10,11] and many others, the RTM method was also used and useful for imaging obstacles in several applications. Studies were conducted using acoustic waves within a free space application in [12], within a planar waveguide in [13], within a half-space in [14], with only intensity measurement in [15]; using electromagnetic waves within free space in [16], within a rectangular waveguide in [17], within an application based only on the intensity of data in [18], within a biomedical tomography at optical frequencies in [19]; and using elastic waves within a half-space in [20].

Detection and imaging of obstacles embedded in a stratified medium find a wide application area including underground imaging and through-wall imaging (TWI) as two-layered and three-layered medium applications, respectively [21,22]. In addition to radar techniques and inversion algorithms [21,22], direct methods to probe a structure stand out as a field of study in which many applications are clustered around [1,2,3,4,5,6,7,8,9,10,11,12,13,14,15,16,17,18,19,20,23,24,25,26,27,28,29,30,31,32,33,34,35].

Applications governed by the scalar wave equation are more feasible due to computational expenses arising as a challenging problem. Employing an asymptotic filter to map the real 3D data, which are obtained on an acquisition surface far enough from the reconstruction domain into a 2D space, creates extra room to reduce that cost to lower levels [23,24]. The spatial conversion filter approach in [23] also underlines the waveform, i.e., the argument of a received signal, whereas the phaseless magnitude approach is of concern in [15,18]. Consequently, each part of the data is proven to have its own benefit.

Despite the fact that both subsurface imaging and TWI are widespread areas of research [2,3,4,14,20,23,24,25,26,27,28,29,30,31], in-wall imaging applications have remained a relatively untouched area. In-wall imaging applications may be divided into three categories:

- Detection and imaging of embedded obstacles in an inaccessible wall [36,37,38,39,40,41];
- Estimating wall parameters [42,43,44,45,46,47];
- Clutter reduction in TWI [48,49,50,51].

Imaging of sparse objects with the Born approximation and the compressive sensing approach is studied in [36]. It was proposed that the hypothesis of sparsity makes it possible to reduce the size of the data. A novel approach using the valuable features of the linear sampling method is presented in [37] for imaging the geometrical properties of an embedded object. A microwave tomographic approach is employed to process ground penetrating radar (GPR) data for detecting and locating defects in a historical wall [38]. A shadow projection method is studied to determine the geometrical properties of air gaps in a dense medium [39]. The shadow projection method is based on the data obtained on the boundary behind the object. The method exploits the lensing effect emerging between the two adjacent layers with high contrast. A prototype based on impulse radio ultra-wideband radar is developed for identifying the current condition of a wall [40]. The prototype includes a deep learning module for a robust and self-adaptive application. Synthetic aperture radar imaging is employed for the reconstruction of the infrastructure elements within a dry wall in [41]. For this purpose, a frequency-modulated continuous-wave radar operating at 80 GHz with a bandwidth of 25.6 GHz is used.

The most basic wall parameters are dielectric permittivity, conductivity, and thickness. Reconstruction of wall parameters is conducted for several reasons such as determining the thickness of a wall, checking its health condition, detecting the infrastructures within, and planning an indoor wireless broadcast scheme [42,43,44,45,46,47].

The signal response of a wall is always stronger than that of a passive object located behind it and therefore a clutter reduction procedure is needed in TWI applications [48,49,50,51].

Based on the RTM method, a radar application is conducted for imaging targets buried in a multilayered underground with the help of probes located inside the test site through a dug hole [32]. For the sake of the structural reliability in some components, imaging of notches and defects on aluminum samples by using an ultrasonic array leaning on the material at zero distance to excite elastic waves is introduced in [33,34]. The internal defect detection of casting steel is studied in [35].

Nondestructive testing techniques constitute a big family. One member of this family is microwave imaging, which uses electromagnetic waves in the microwave frequency band [52,53,54]. Electromagnetic illumination is achieved by using this frequency band throughout this study.

The main aim of this paper is to produce fundamental results for in-wall imaging with the RTM method for the detection and imaging of embedded obstacles at microwave frequencies. Within this context, imaging an embedded object in the inaccessible middle layer of a three-layered medium is considered.

The rest of the study presented here is organized as follows. In Section 2, the geometry of the problem and the general formulation are given. In Section 3, the RTM method based on obtaining support within a reconstruction domain is presented and the application guidelines of the Monte Carlo simulation within this study are introduced. In Section 4, a comprehensive application through a properly defined set of numerical experiments is conducted to observe the performance of the algorithm for in-wall imaging. Additionally, the outline of the quantitative performance evaluation is included and discussed in this section. Section 5 is the Conclusion section revealing a summary as concluding remarks for the study.

Wave excitation is achieved under the time-harmonic regime. To this end, a time factor $e^{-i\omega t}$ is selected to be dropped in this paper including the whole numerical experiments conducted and presented in Section 4.

## 2. Formulation of the Problem

**Figure 1:**
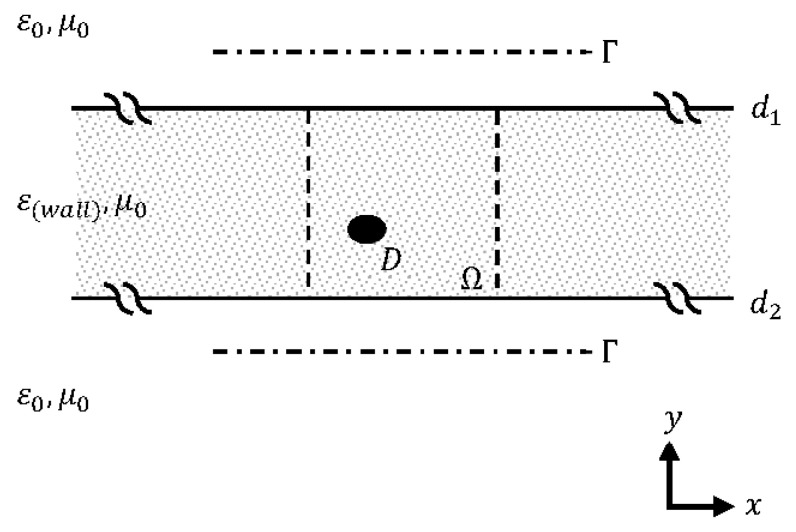
Geometry of the problem.

The reference geometry of the problem considered here is a wall structure of finite thickness laying along the horizontal axis, basically a three-layered medium configuration with the object of interest sitting on a finite portion of it. That geometry is depicted in Figure 1. The wall is defined as a lossless material with a relative permittivity $\varepsilon_{r_{2}}=\varepsilon_{r\left(wall\right)}$ and a thickness $\left|d_{1}-d_{2}\right|$. The first and the third layers are assumed to be free space, $\varepsilon_{r_{1}}=\varepsilon_{r_{3}}=1$. The magnetic permeability is the same as that in free space, $\mu_{r}=1$, in every area. The reconstruction domain, denoted by Ω, is a subset of the wall being the region of interest that the object (domain), D, is located within. Time-harmonic field excitations are achieved by infinite line sources and a 2D geometry is considered. Locations of the source and the observation points are selected to be identical and they are positioned on both or one of the two sides of the wall forming a multibistatic configuration for data acquisition, which is denoted as Γ.

Wave dynamics in the presence of an infinite line (a TM polarized) source is described by the Helmholtz wave equation as:

(1) $$\Delta E+k^{2}E=-i\omega \mu l\cdot \delta (x-x_{0})\cdot \delta (y-y_{0}),$$

where E=E(x,y) denotes the electric field deviation; k is the wavenumber; ω is the operating frequency; μ is the magnetic permeability in the medium; I is the amplitude of the electric current source; (x,y) is the cartesian coordinate system denoting points of field (observation); and $\left(x_{0},y_{0}\right)$ is the point of a line source excitation [55]. The Helmholtz equation is described as a differential operator acting on a field distribution and the solution is given by the (scalar) Green’s function, G, as:

(2) $$\Delta G\left(x,y;x_{0},y_{0}\right)+k^{2}G\left(x,y;x_{0},y_{0}\right)=-\delta \left(x-x_{0}\right)\cdot \delta \left(y-y_{0}\right),$$

resulting in a fundamental solution to Equation (1) under the conditions determined due to the boundaries of the layers at a problem geometry of interest as:

(3) $$G\;\mathrm{and}\;\frac{\partial G}{\partial y}\;\mathrm{are}\;\mathrm{continuous}\;\mathrm{on}\;\mathrm{the}\;\mathrm{boundaries},$$

with the radiation condition as the distance from the excitation point, $\left|\vec{\rho }-{\vec{\rho }}_{0}\right|$, tending to infinity [55]. Here, $\vec{\rho }=\left(\rho ,\phi \right)\equiv \left(x,y\right)$ denotes the polar coordinate system and ${\vec{\rho }}_{0}=\left(\rho_{0},\phi_{0}\right)\equiv \left(x_{0},y_{0}\right)$ is the point of the line source excitation.

The solution of Equations (2) and (3) for $G\left(x,y;x^{\prime },y^{\prime }\right)$ is given as an explicit expression due to the problem geometry and is explained in detail in the literature for many cases including free space and multilayered media geometries [55,56,57,58]. Under the time convention given in this paper, the Hankel function of the first kind, $H_{0}^{\left(1\right)},$ is reserved for outgoing waves and of the second kind, $H_{0}^{\left(2\right)}$, is for incoming ones. In free space, Green’s function expression is given as follows:

(4) $$G\left(x,y;x^{\prime },y^{\prime }\right)=\frac{i}{4}\cdot H_{0}^{\left(1\right)}\left(k_{0}\left|\vec{\rho }-{\vec{\rho }}_{0}\right|\right).$$

where $k_{0}$ denotes the wavenumber of the free space.

In a three-layered medium, the problem geometry is more complicated and the expression of $G\left(x,y;x^{\prime },y^{\prime }\right)$ occupies considerable space. To this end, all expressions are given in Appendix A.

The pair of scattering equations for the field scattered from a dielectric object, $E^{s}$, due to an incident field, $E^{i}$, are described by those two Fredholm integral equations as [55,56,57,58].

(5) $$E^{s}\left(x,y\right)=k^{2}\overset{}{\underset{D\left[\Omega \right]}{\iint }}\chi \left(x^{\prime },y^{\prime }\right)E\left(x^{\prime },y^{\prime }\right)G\left(x,y;x^{\prime },y^{\prime }\right)dx^{\prime }dy^{\prime }\;\;\;;\;\;\;\left(x,y\right)\in \Gamma \;\mathrm{and}\;\left(x^{\prime },y^{\prime }\right)\in D\left[\Omega \right],$$

(6) $$E\left(x,y\right)=E^{i}\left(x,y\right)+k^{2}\overset{}{\underset{D\left[\Omega \right]}{\iint }}\chi \left(x^{\prime },y^{\prime }\right)E\left(x^{\prime },y^{\prime }\right)G\left(x,y;x^{\prime },y^{\prime }\right)dx^{\prime }dy^{\prime }\;\;\;;\;\;\;\left(x,y\right),\;\left(x^{\prime },y^{\prime }\right)\in D\left[\Omega \right],$$

where (x,y) are the points on a selected acquisition line; $\left(x^{\prime },y^{\prime }\right)$ are the points on the object domain or the reconstruction domain; and $\chi ={\hat{\varepsilon }}_{r}\left(x,y\right)-1$ is the object’s function with ${\hat{\varepsilon }}_{r}$ denoting the complex permittivity. k is the wavenumber of that layer at which the object domain is located. E is the total electric field inside the reconstruction domain and is described by the implicit expression in Equation (6) [55,56,57,58].

Similarly, the field scattered from a perfect electric conductor (PEC) object is described by another pair as follows [55,56,57,58]:

(7) $$E^{s}\left(x,y\right)=\overset{}{\underset{\partial D}{\int }}j\left(x^{\prime },y^{\prime }\right)E^{i}\left(x,y;x^{\prime },y^{\prime }\right)dl^{\prime };\left(x,y\right)\in \Gamma \;\mathrm{and}\;\left(x^{\prime },y^{\prime }\right)\in \partial D,$$

(8) $$E\left(x,y\right)=E^{i}\left(x,y\right)+E^{s}\left(x,y\right)=0\;\mathrm{on}\;\partial D.$$

Here, $\left(x^{\prime },y^{\prime }\right)$ are the points located on the surface of the PEC object. ∂D denotes the boundary of the object domain, D. j and E denote the inducted electric current deviation and the total electric field on that surface, respectively. The expression given in Equation (8) is, in fact, another form of Equation (3) due to the presence of a PEC discontinuity [55,56,57,58].

## 3. The Method

### 3.1. Reverse Time Migration (RTM) Method

By obtaining the back-propagation and computing the cross-correlation, the RTM method employed here is based on creating support for obstacles embedded in a medium by using the far-field scattering data, $E^{s}$, [12,13,14,15,16,17,18,19,20]. It is assumed that there are $N_{s}$ and $N_{r}$ locations on $\Gamma_{s}=\partial B_{s}$ and $\Gamma_{r}=\partial B_{r}$ as points of source and observation, respectively. $B_{s}$ and $B_{r}$ denote continuous line segments, open or closed curves where $N_{s}$ points of source and $N_{r}$ points of observation are located at some points of them, respectively: $\Gamma_{s,r}=\partial B_{s,r}\subset B_{s,r}$. The reconstruction domain may fall inside both or one of those two with obstacles in it, or may not.

The back-propagation, $E^{b}$, is equivalent to the propagation excited by the time-reversed form of the scattered data which is the complex conjugation in the frequency domain. The time-reversed scattered data stand as a source term in the Helmholtz equation that will be solved for back-propagation [12,13,14,15,16,17,18,19,20]:

(9) $$\Delta u^{b}\left(x,y;x_{s},y_{s}\right)+k^{2}u^{b}\left(x,y;x_{s},y_{s}\right)=\frac{\left|\Gamma_{r}\right|}{N_{r}}\cdot \overset{N_{r}}{\underset{r=1\;}{\sum }}\bar{u^{s}\left(x_{r},y_{r};x_{s},y_{s}\right)}\delta \left(x-x_{r}\right)\delta \left(y-y_{r}\right),$$

(10) $$\left|\vec{\rho }-{\vec{\rho }}_{r}\right|\to \infty ;\sqrt{\left|\vec{\rho }-{\vec{\rho }}_{r}\right|}\left(\frac{\partial u^{b}}{\partial \left|\vec{\rho }-{\vec{\rho }}_{r}\right|}-iku^{b}\right)\to 0,$$

(11) $$u^{b}\left(x,y;x_{s},y_{s}\right)=-\frac{\left|\Gamma_{r}\right|}{N_{r}}\cdot \overset{N_{r}}{\underset{r=1\;}{\sum }}G\left(x,y;x_{r},y_{r}\right)\bar{u^{s}\left(x_{r},y_{r};x_{s},y_{s}\right)}.$$

Here, $\left(x_{s},y_{s}\right)$ and $\left(x_{r},y_{r}\right)$ denote the points of source and observation on $\Gamma_{s}$ and $\Gamma_{r}$, respectively, and (x,y) denotes any point on a continuous reconstruction domain. $\frac{\left|\Gamma_{r}\right|}{N_{r}}$ is placed in Equations (9) and (11) for stabilization and $\left|\Gamma_{r}\right|$ denotes the length of $\Gamma_{r}$. $u^{s}$ and $u^{b}$ are the fundamental expressions of the scattered field and the back-propagation, respectively: $E^{s}=i\omega \mu I\cdot u^{s}$ and $E^{b}=i\omega \mu I\cdot u^{b}$. $\bar{u^{s}}$ denotes the complex conjugation of $u^{s}$.

Hence the cross-correlation, namely the support, is computed between the back-propagation and the incident fields emitted from sources on $\Gamma_{s}$. It is described as the inner product of those two quantities [12,13,14,15,16,17,18,19,20]. Under finite $N_{s}$ and $N_{r}$, the explicit expression of the support function is given as [12,13,14,15,16,17,18,19,20]:

(12) $$I\left(x,y\right)=-k^{2}\cdot \frac{\left|\Gamma_{s}\right|\cdot \left|\Gamma_{r}\right|}{N_{s}\cdot N_{r}}\cdot \overset{N_{s}}{\underset{s=1}{\sum }}\overset{N_{r}}{\underset{r=1\;}{\sum }}G\left(x,y;x_{s},y_{s}\right)G\left(x,y;x_{r},y_{r}\right)\bar{u^{s}\left(x_{r},y_{r};x_{s},y_{s}\right)}\;\;\;;\;\;\;\left(x_{s,r},y_{s,r}\right)\in \Gamma_{s,r}\;and\;\left(x,y\right)\in \Omega ,$$

Here, $\frac{\left|\Gamma_{s}\right|\cdot \left|\Gamma_{r}\right|}{N_{s}\cdot N_{r}}$ is placed in Equation (12) for stabilization and $\left|\Gamma_{s}\right|$ denotes the length of $\Gamma_{s}$.

The formulation of the algorithm is independent of any a priori information on the physical properties of the embedded object [12,13,14,15,20].

### 3.2. Monte Carlo Simulation

Reporting the level of achievement of the RTM method in in-wall imaging based on a single test would not be fair and would also be misleading. In order to make a general inference, the method should be tested under different circumstances. The Monte Carlo simulation scheme can be adapted for this purpose [59]. A properly defined set of experiments may be employed in the outline of this approach within a comparative study. Each experiment results in a different level of achievement and an overall evaluation will be possible by combining all of them together. Then, it is possible to make a proper conclusion.

In order to achieve this goal, first, a set of parameters is defined. Those parameters represent the domain of possible inputs in the Monte Carlo simulation scheme and they are listed as follows:

1. Acquisition line;
2. Material type of the embedded object;
3. Structural property of the embedded object;
4. Location of the embedded object;
5. Operating wavelength;
6. Noise level in the medium.

Any change in any parameter results in a different experiment with different scattered data. The scattered data, $u^{s}\left(x_{r},y_{r};x_{s},y_{s}\right)$, is synthetically obtained from the pair of scattering equations given in Equations (5) and (6) for a dielectric object and in Equations (7) and (8) for a PEC object. Hence, the noise level in the medium is added to the scattered data. The Green’s functions are $G\left(x,y;x_{s},y_{s}\right)$ and $G\left(x,y;x_{r},y_{r}\right)$ and they are synthetically obtained from Equation (4) or (A1)–(A13). In the Monte Carlo simulation scheme, the scattered data and Green’s functions are taken instead of randomly generated inputs. The deterministic computation step is defined in Equation (12). After all of those, the level of achievement is calculated quantitatively for each experiment to make a final assessment.

**Figure 2:**
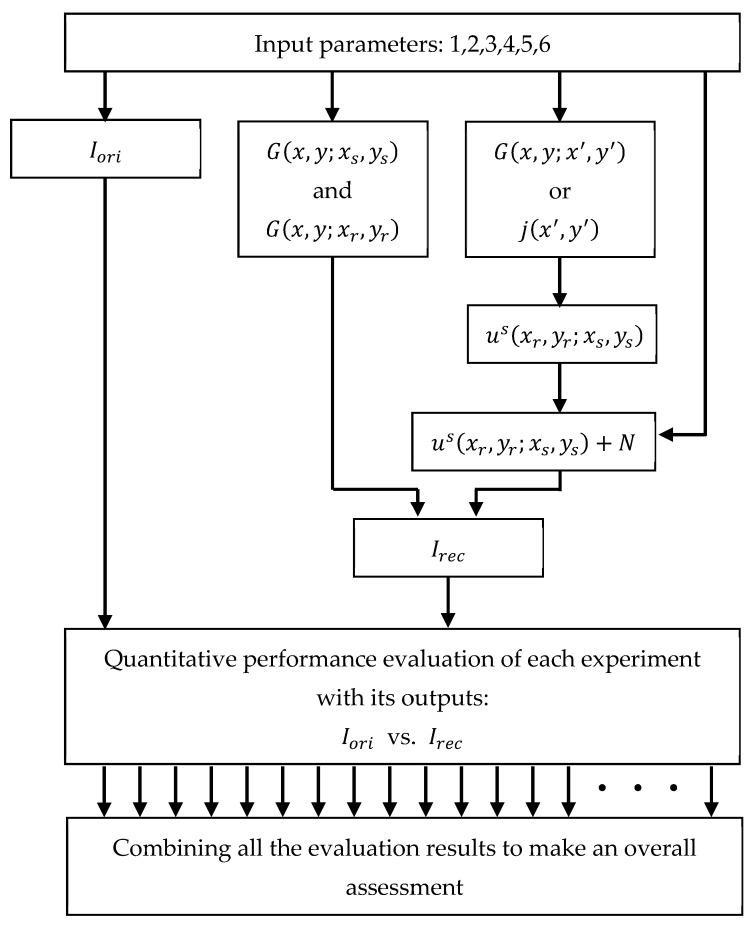
The block diagram of the Monte Carlo simulation scheme adapted for the RTM method.

The block diagram of the Monte Carlo simulation scheme adapted for the RTM method is given in Figure 2.

The blocks of the diagram in Figure 2 are described as follows:

- Input parameters are the six parameters listed above;
- $I_{ori}$ is the original information data;
- $G\left(x,y;x_{s},y_{s}\right)$ and $G\left(x,y;x_{r},y_{r}\right)$ are the Green’s functions computed on $\left(\Omega ;\Gamma_{s}\right)$ and $\left(\Omega ;\Gamma_{r}\right)$, respectively;
- If the embedded object is a dielectric material, Green’s functions are computed on two different domains, $G\left(x,y;x^{\prime },y^{\prime }\right)$ on $\left(\Gamma_{r};D\right)$ and (D;D);
- If it is a PEC material, the inducted electric current is computed on the surface of the object, $j\left(x^{\prime },y^{\prime }\right)$ on ∂D;
- $u^{s}\left(x_{r},y_{r};x_{s},y_{s}\right)$ is the scattered data synthetically acquired on $\Gamma_{r}$ due to the sources located at $\Gamma_{s}$;
- The noise level in the medium is added to the noise-free data: $u^{s}\left(x_{r},y_{r};x_{s},y_{s}\right)+N$;
- $I_{rec}$ is the reconstructed information data obtained from the RTM method;
- Procedures for quantitative performance evaluation of an experiment and an overall assessment of the whole experiment set are considered in Section 4.

## 4. Numerical Experiments and Discussion

In order to show the performance of the algorithm in line with reconstructing objects embedded in a wall, part of which is equivalent to those applications given in [12,16]. In other words, those examples of adaptation to in-wall imaging are considered by copying some of the parameters except those determined by the problem geometry. For instance, points of source and observation cannot be located inside the wall within the sense of complete nondestructive testing and using an additional probe inside the wall is not considered. Thus, it will be possible to establish a comparative study.

Covering the range y=(−3,+3) m, there is a wall of thickness at 6 m and it lies along the whole Ox axis. The relative permittivity of the wall is $\varepsilon_{r\left(wall\right)}=2$. The reconstruction domain covers some limited part of that wall as it extends within the range of Ω=(−3,+3) m×(−3,+3) m. The domain, Ω, is laid out on a grid pattern of 200×200 meshes of equal size and the correlation functionals are computed at their central points [56].

A set of experiments, each touching upon a different aspect of the subject, is created to acquire different performance outputs of the method due to different situations.

The first parameter is the acquisition line. Four different sets of probes are used:

- The first set, $\Gamma_{1}=\partial B_{1}$, is formed by 97 points of source and observation uniformly located within the range x=(−6,+6) m on one side of the wall at y=+4 m and the same on the other side at y=−4 m being $N_{1}=97+97=194$ in total illuminating the area of the size $12\;m\times 8\;m=96{\;m}^{2}$. With this set, it is possible to perform a double-sided illumination and measurement;
- In the second and third sets, $\Gamma_{2}$ and $\Gamma_{3}$, are formed by using only the upper half and the lower half of the first set located at the same line segments at y=+4 m and y=−4 m, respectively: $\Gamma_{2,3}=\partial B_{2,3}$ and $N_{2,3}=97$. One-sided illumination and measurement is performed in case one of the two sides of a wall is inaccessible;
- There is also a zeroth set, $\Gamma_{0}=\partial B_{0}$, formed by different points of source and observation, which are uniformly distributed on a circle with radius $r=\sqrt{\frac{12\times 8}{\pi }}\approx 5.53\;m$ and $N_{0}=194$ illuminating the same size of the area as that in the first set and it is given only in the absence of the wall for comparison.

The reconstruction domain, Ω, is surrounded by both the first and the zeroth sets and sits in the middle of both those two different closed curves, $B_{0}$ and $B_{1}$.

Points of fully isotropic source and observation uniformly located on a circle with a radius big enough to encapsulate the reconstruction domain in an unbounded medium from all directions (here, it is $\Gamma_{0}$) stands as a reference level of perfection for data acquisition and using another acquisition line instead mostly leads to some imperfections in reconstruction [16]. In a non-destructive in-wall imaging application, there are some restrictions on determining such a proper acquisition line as it lacks that kind of perfect surrounding property. There cannot be any penetration into the wall, therefore it ends up with some probable imperfection, as expected [16].

The second parameter is the material type. The object may be selected from three different types of materials:

- First, a dielectric object of penetrable material with a contrast ratio of $\varepsilon_{r\left(object\right)}/\varepsilon_{r\left(medium\right)}=2$;
- Additionally, an air gap only in the presence of the wall, again, as a penetrable material, with a contrast ratio of $\varepsilon_{r\left(object\right)}/\varepsilon_{r\left(wall\right)}=0.5$;
- Thirdly, a PEC object of non-penetrable material.

The third parameter is the structural property of the embedded object. Based on this, two canonic geometries are selected:

- A unit circle, bigger in size carrying a basic curvature property;
- A point body that stands for smaller objects being poor in structural properties.

The fourth parameter is the location. The object may be located at four different positions:

- In the center or the middle point of the reconstruction domain, (x,y)=(0,0) m;
- In an unbalanced location in the horizontal direction, (x,y)=(−1,0) m;
- In an unbalanced location in the vertical direction, (x,y)=(0,−1) m;
- In a fully unbalanced location in both the horizontal and the vertical directions, (x,y)=(−1,−1) m.

According to the grid structure, the point body is a single mesh located at (x,y)=(−0.03;−0.03)−(0;0) m, (−1.02;−0.03)−(−0.99;0) m, (−0.03;−1.02)−(0;−0.99) m, and (−1.02;−1.02)−(−0.99;−0.99) m in the first, second, third, and fourth cases, respectively, whereas the unit circle is always centered at one of those four locations listed above.

The fifth parameter is the operating wavelength and three different values are selected: λ=1 m, 0.5 m, and 0.25 m.

The sixth parameter is the noise level and four different noise levels are selected: N ~ 0%, 10%, 20%, and 50%.

There were 1632 numerical experiments conducted. In total, 768 of them were free space applications and the remaining 864 were conducted in the presence of a wall. There were 96 more than the initial 768 since it is also another option to locate or embed material into a wall producing contrast in the reverse order, i.e., a material of some permittivity less than that of the medium, which here was an air gap. One could find out that each of the 192 experiments of those 768 is conducted with $\Gamma_{0}$, $\Gamma_{1}$, $\Gamma_{2}$, and $\Gamma_{3}$, and each of those 288 from the remaining 864, which is again 96 more, is carried out with $\Gamma_{1}$, $\Gamma_{2}$, and $\Gamma_{3}$, respectively. Any change in any parameter reveals a different experiment. By combining them all together, an essential multiparameter Monte Carlo simulation is established through all those experiments for the assessment of an overall fundamental performance evaluation of the method in in-wall imaging applications.

**Figure 3:**
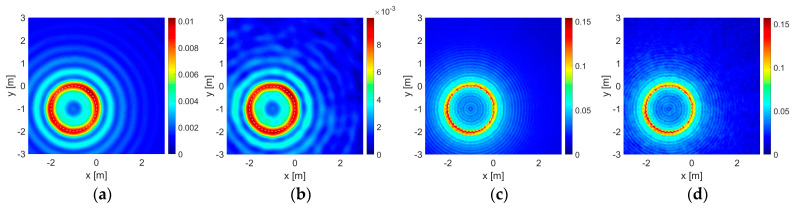

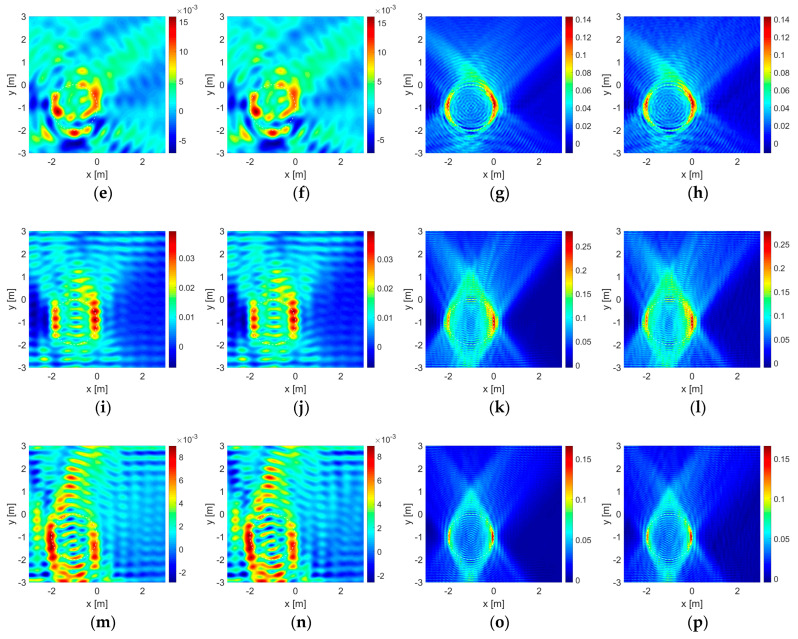
Reconstructions of the dielectric unit circle as the contour plots of correlation functionals: (**a**–**d**) by $\Gamma_{0}$ in free space, (**e**–**h**) by $\Gamma_{1}$ in free space, (**i**–**l**) by $\Gamma_{1}$ in the presence of the wall. (**m**–**p**) Reconstructions of the air gap unit circle by $\Gamma_{1}$ in the presence of the wall. Operating wavelength is λ=1 m in the first and the second columns (**a**,**b**,**e**,**f**,**i**,**j**,**m**,**n**) and λ=0.25 m in the third and the fourth columns (**c**,**d**,**g**,**h**,**k**,**l**,**o**,**p**). Reconstructions with the noise-free data are given in the first and the third columns (**a**,**c**,**e**,**g**,**i**,**k**,**m**,**o**) and those with an additional uniform noise level of 50% are in the second and the fourth columns (**b**,**d**,**f**,**h**,**j**,**l**,**n**,**p**). The color bars show the value distribution of the imaginary part of the function I(x,y) on the reconstruction domain for each case. The horizontal and the vertical axes show the boundaries of the reconstruction domain: Ω=(−3,+3) m×(−3,+3) m.

**Figure 4:**
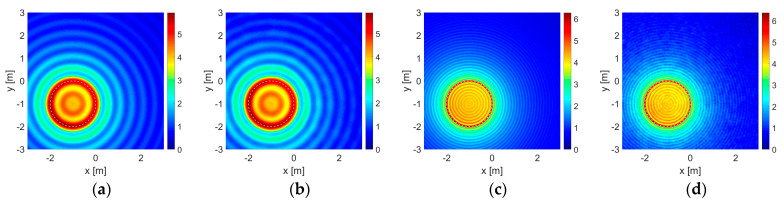

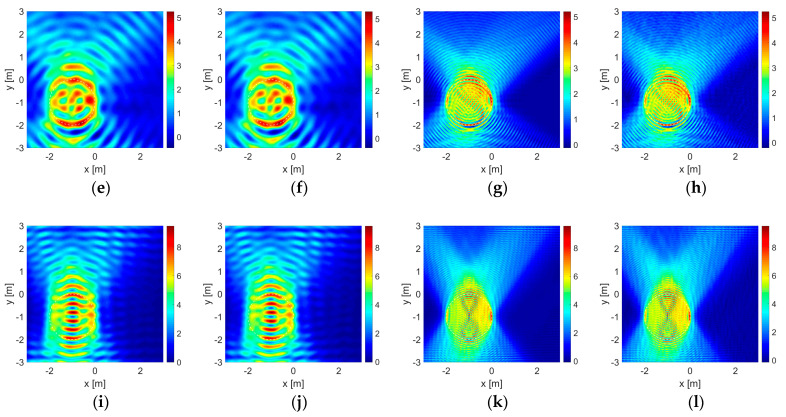
Reconstructions of the PEC circle are given in (**a**–**l**) in the same order as in Figure 3.

**Figure 5:**
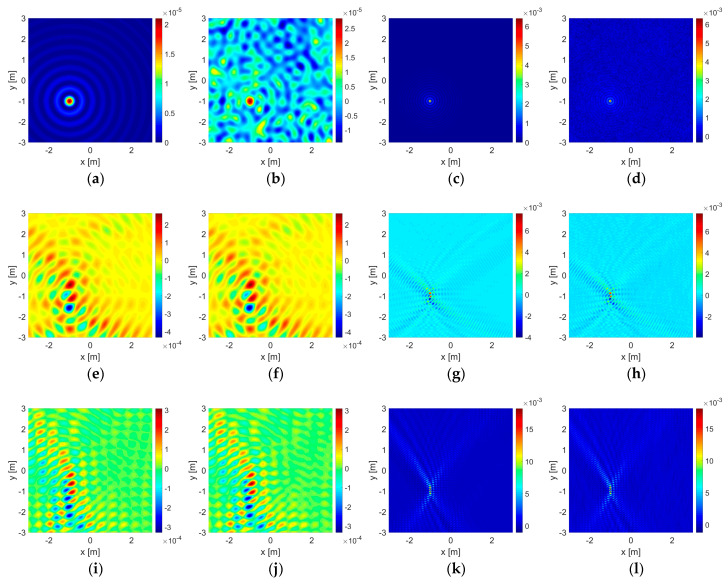

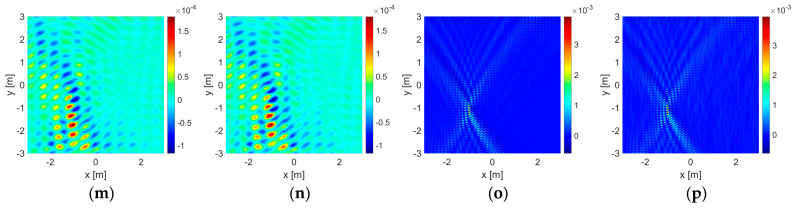
Reconstructions of the dielectric and the air gap point bodies are given in (**a**–**p**) within the same order as in Figure 3.

**Figure 6:**
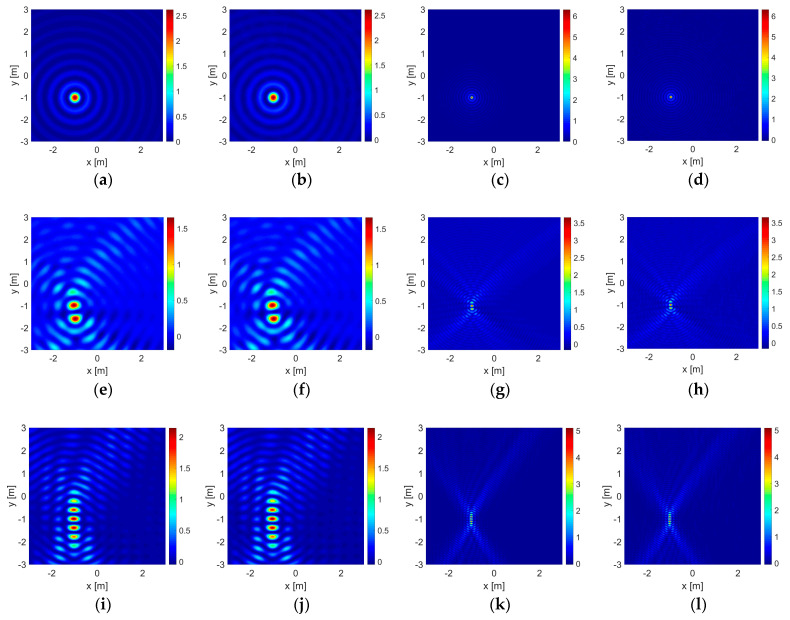
Reconstructions of the PEC point body are given in (**a**–**l**) in the same order as in Figure 4.

The results for some of those cases in which the objects are located at the most unbalanced position are given in Figure 3 and Figure 4 for the dielectric and the PEC unit circles, and in Figure 5 and Figure 6 for the dielectric and the PEC point bodies, respectively. The plots are given only for the imaginary parts of the correlation functionals [12,13,14,15,16,17,18,20] due to sign compliance [12,13,16]. In Figure 3, in the first row, the dielectric unit circle reconstruction with $\Gamma_{0}$ is given in the absence of the wall for comparison. Reconstruction of the same object with the double-sided multibistatic configuration, $\Gamma_{1}$, again, in the absence of the wall for comparison and also in the presence of it are given in the second and the third rows, respectively. Reconstruction of the air gap is given in the fourth row. The PEC unit circle reconstructions are given in Figure 4 in the same order as in Figure 3. The point body reconstructions are given in Figure 5 and Figure 6 in the same order as in Figure 3 and Figure 4.

Such contour plots placed side-by-side, as those given in Figure 3, Figure 4, Figure 5 and Figure 6, present instances for visual comparisons [60]. However, it is hard to state that visual comparison is suitable for an overall evaluation of the whole of the 1632 experiments and the outputs. Mean squared error (MSE) of the support functions, $I_{rec}$, with respect to the original data, $I_{ori}$, may facilitate an efficient quantitative evaluation. Therefore, a proper definition must be made for modeling the original data. A mesh on a given grid pattern with more than 50% of it covered by part of the object is valued at 1, otherwise, it is 0. Additionally, a PEC object does not allow electromagnetic waves to penetrate inside the region bounded by itself and therefore they are stuffed materials whereas dielectric objects are penetrable. Therefore both PEC circles and disks of the same size are taken as stuffed and identical to each other. On the other hand, dielectric circles and disk structures are different due to wave penetration and a dielectric circle is taken as empty or hollow. Suffering from dimension, there is no such discussion on point bodies.

Working with raw data for the calculation of MSE values is similar to comparing apples with oranges. Using the min–max scaling, a normalization procedure is followed for linearly mapping the values of the support functions into the range [0, 1]. Denoting the normalized data as ${\hat{I}}_{rec}$, the MSE calculation for $I_{ori}$ and ${\hat{I}}_{rec}$ instead of $I_{ori}$ and $I_{rec}$ is carried out and the quantitative performance evaluation percent, P, is given in the outline of this definition, accordingly:

(13) $$P\left(I_{ori},I_{rec}\right)=\left[1-\mathrm{MSE}\left(I_{ori},{\hat{I}}_{rec}\right)\right]\times 100.$$

It is important to note that as the area of a reconstruction domain tends to infinity, any embedded object becomes a truly dimensionless point body and the MSE value approaches a perfect value of 0. This means that the MSE calculation can only be of use in a comparative study within a finite reconstruction domain in order to make a proper evaluation. As a consequence of this, it may be expected that the MSE calculation would be in favor of the point body reconstructions instead of those conducted with a unit circle since a unit circle is bigger in size than a point body. On the other hand, bigger objects return greater responses on the same acquisition line. The magnitude values of a support function on those meshes at which part of the object is located are mostly much greater than the rest and one may look that situation up in Figure 3, Figure 4, Figure 5 and Figure 6. As a result of those two, the formulation proposed in (13) becomes reasonable for a quantitative performance evaluation.

In line with the parameters listed above, the Monte Carlo simulation outputs are given to show the performance levels by acquisition line, material property, structural property, object location, operating wavelength, and noise level. The results are given in a crosstab form in Table 1, Table 2, Table 3, Table 4, Table 5 and Table 6, respectively. In the first row of the tables, those experiments conducted with the acquisition line $\Gamma_{0}$ are given in the absence of the wall for comparison. The reconstructions with $\Gamma_{1}$, $\Gamma_{2}$, and $\Gamma_{3}$, again, in the absence of the wall for comparison and also in the presence of it are given in the second and the third rows, respectively. On the far right, the overall evaluation is always placed in bold issuing a condensed brief of the study.

In Table 6, the robustness of the algorithm is shown due to different levels of additive uniform noise, which is also presented for visual comparison in Figure 3, Figure 4, Figure 5 and Figure 6.

The presence of a wall has veiling and bending effects on electromagnetic waves. As an electromagnetic wave hits a wall, some portion of it does not penetrate into it to reflect back, resulting in a reduction in signal power in the first place. On the other hand, the wall triggers multiple reflections and transmissions in the medium and a series of interactions emerges between the (boundaries of the) wall and the embedded object increasingly adding some extra power to the signal strength. This situation is also seen in the value scales of the contour plots in Figure 3, Figure 4, Figure 5 and Figure 6. Overall results show that, in most cases, those interactions gain an advantage and have constructive effects on reconstruction performance in the end.

## 5. Conclusions

A correlation-based algorithm, the RTM method, is studied for the reconstruction of objects within an inaccessible wall. A comparative study is presented. Numerical experiments put forth essential achievements giving promising results for further studies and making it feasible for in-wall imaging applications.

Inevitably, using a set of probes located outside of the wall instead of perfectly surrounding the reconstruction domain deforms the reconstruction. Interactions between the embedded obstacle and the boundaries due to the presence of a wall geometry occur to work as a retouching tool in the horizontal direction and have positive effects on the reconstruction. On the other hand, those interactions produce interferences deforming the reconstruction in the vertical direction. As the distance of an object away from the boundaries of the wall increases/decreases, it also improves/deteriorates the imaging quality. With a decrease in operating wavelength or at higher operating frequencies, that distance is relatively on the rise.

Much more complicated problem geometries and real-life applications may be taken into consideration for future works in order to see where the efficiency and the applicability levels of the method could reach further in in-wall imaging.

## Figures and Tables

**Table 1:** Performance by acquisition line.

	Perfectly Surrounding $\Gamma_{0}$	Double-Sided $\Gamma_{1}$	One-Sided Upper|Lower $\Gamma_{2}\;|\;\Gamma_{3}$	Overall	
with $\Gamma_{0}$ in the absence of the wall	96.78	−	−	**96.78**	%
with $\Gamma_{1,2,3}$ in the absence of the wall	−	89.27	75.55 | 76.40	**82.62**	%
with $\Gamma_{1,2,3}$ in the presence of the wall	−	92.15	75.07 | 75.37	**83.68**	%

**Table 2:** Performance by material property of the embedded object.

	Dielectric	Air Gap	PEC	Overall	
with $\Gamma_{0}$ in the absence of the wall	96.24	−	97.33	**96.78**	%
with $\Gamma_{1,2,3}$ in the absence of the wall	80.11	−	85.14	**82.62**	%
with $\Gamma_{1,2,3}$ in the presence of the wall	82.02	82.51	86.52	**83.68**	%

**Table 3:** Performance by structural property of the embedded object.

	Unit Circle	Point Body	Overall	
with $\Gamma_{0}$ in the absence of the wall	95.06	98.51	**96.78**	%
with $\Gamma_{1,2,3}$ in the absence of the wall	83.63	81.62	**82.62**	%
with $\Gamma_{1,2,3}$ in the presence of the wall	83.47	83.90	**83.68**	%

**Table 4:** Performance by object location.

	(0,0) m	(−1,0) m	(0,−1) m	(−1,−1) m	Overall	
with $\Gamma_{0}$ in the absence of the wall	96.85	96.73	96.61	96.94	**96.78**	%
with $\Gamma_{1,2,3}$ in the absence of the wall	82.30	83.15	82.49	82.56	**82.62**	%
with $\Gamma_{1,2,3}$ in the presence of the wall	83.65	83.90	83.62	83.56	**83.68**	%

**Table 5:** Performance by operating wavelength.

	λ=1 m	λ=0.5 m	λ=0.25 m	Overall	
with $\Gamma_{0}$ in the absence of the wall	95.03	97.51	97.81	**96.78**	%
with $\Gamma_{1,2,3}$ in the absence of the wall	79.70	82.46	85.71	**82.62**	%
with $\Gamma_{1,2,3}$ in the presence of the wall	81.04	84.21	85.80	**83.68**	%

**Table 6:** Performance by noise level.

	0%	10%	20%	50%	Overall	
with $\Gamma_{0}$ in the absence of the wall	97.55	97.33	96.90	95.34	**96.78**	%
with $\Gamma_{1,2,3}$ in the absence of the wall	82.65	82.64	82.63	82.58	**82.62**	%
with $\Gamma_{1,2,3}$ in the presence of the wall	83.73	83.71	83.69	83.60	**83.68**	%

## Data Availability

The data presented in this study are available on request from the corresponding author.

## Abbreviations

The following abbreviations are used in this manuscript:

RTM	reverse time migration
TWI	through-wall imaging
3D	three-dimensional
2D	two-dimensional
GPR	ground penetrating radar
TM	transverse magnetic
PEC	perfect electric conductor
MSE	mean squared error
SIP	Sommerfeld integration path

## Appendix A

The explicit expression of the Green’s function of a three-layered medium depicted in Figure 1 is given as follows [55,56,57,58]:

(A1) $$G\left(x,y;x^{\prime },y^{\prime }\right)=\left\{\begin{array}{c} G_{11}\left(x,y;x^{\prime },y^{\prime }\right)\;;\;d_{1}<y,y^{\prime }\\ G_{12}\left(x,y;x^{\prime },y^{\prime }\right)\;;\;d_{2}<y<d_{1}\vee d_{1}<y^{\prime }\\ G_{13}\left(x,y;x^{\prime },y^{\prime }\right)\;;\;y<d_{2}\vee d_{1}<y^{\prime }\\ G_{21}\left(x,y;x^{\prime },y^{\prime }\right)\;;\;d_{1}<y\vee d_{2}<y^{\prime }<d_{1}\\ G_{22}\left(x,y;x^{\prime },y^{\prime }\right)\;;\;d_{2}<y,y^{\prime }<d_{1}\\ G_{23}\left(x,y;x^{\prime },y^{\prime }\right)\;;\;y<d_{2}\vee d_{2}<y^{\prime }<d_{1}\\ G_{31}\left(x,y;x^{\prime },y^{\prime }\right)\;;\;d_{1}<y\vee y^{\prime }<d_{2}\\ G_{32}\left(x,y;x^{\prime },y^{\prime }\right)\;;\;d_{2}<y<d_{1}\vee y^{\prime }<d_{2}\\ G_{33}\left(x,y;x^{\prime },y^{\prime }\right)\;;\;y,y^{\prime }<d_{2} \end{array}\right.,$$

(A2) $$G_{mn}\left(x,y;x^{\prime },y^{\prime }\right)=\{\begin{array}{c} \;\frac{i}{4}\cdot H_{0}^{\left(1\right)}\left(k_{m}\left|\vec{\rho }-{\vec{\rho }}^{\prime }\right|\right)\;\;\;;\;\;\;m=n\\ \;0\;\;\;\;\;\;\;\;\;\;\;\;\;\;\;\;\;\;\;\;\;\;\;\;\;\;\;\;\;\;\;\;\;\;\;\;\;\;\;;\;\;\;m\neq n \end{array}\;+\frac{i}{4\pi }\cdot \overset{+\infty }{\underset{\begin{array}{c} -\infty \\ \left(SIP\right) \end{array}}{\int }}\frac{1}{k_{my}}\cdot e^{ik_{x}\left(x-x^{\prime }\right)}\cdot g_{mn}\cdot dk_{x},$$

(A3) $$k_{my}=\sqrt{k_{m}^{2}-k_{x}^{2}}\;\;\;;\;\;\;m=1,2,3.$$

Here, $k_{m}$ denotes the wavenumber of the mth layer. The first and the third layers are free space and the middle layer is the wall structure: $k_{1}=k_{3}=k_{0}$ and $k_{2}=k_{\left(wall\right)}$. SIP denotes the Sommerfeld integration path. The parameter denoted as $g_{mn}$ in the integrand in Equation (A2) is defined due to the vertical positions of source and observation points as follows:

(A4) $$\begin{array}{c} g_{11}=e^{ik_{1y}\left(y+y^{\prime }\right)}\cdot e^{-2ik_{1y}d_{1}}\cdot \frac{k_{1y}-k_{2y}}{k_{1y}+k_{2y}}\\ +e^{ik_{1y}\left(y+y^{\prime }\right)}\cdot e^{2i\left(-k_{1y}d_{1}+k_{2y}d_{1}-k_{2y}d_{2}\right)}\cdot 4k_{1y}k_{2y}\cdot \frac{k_{2y}-k_{3y}}{k_{1y}+k_{2y}}\cdot \frac{1}{\gamma }, \end{array}$$

(A5) $$\begin{array}{c} g_{12}=e^{i\left(k_{1y}y^{\prime }-k_{2y}y\right)}\cdot e^{i\left(k_{2y}-k_{1y}\right)d_{1}}\cdot 2k_{1y}\cdot \left(k_{2y}+k_{3y}\right)\cdot \frac{1}{\gamma }\\ +e^{i\left(k_{1y}y^{\prime }+k_{2y}y\right)}\cdot e^{i\left(k_{2y}-k_{1y}\right)d_{1}}\cdot e^{-2ik_{2y}d_{2}}\cdot 2k_{1y}\cdot \left(k_{2y}-k_{3y}\right)\cdot \frac{1}{\gamma }, \end{array}$$

(A6) $$g_{13}=e^{i\left(k_{1y}y^{\prime }-k_{3y}y\right)}\cdot e^{i\left(k_{3y}-k_{2y}\right)d_{2}}\cdot e^{i\left(k_{2y}-k_{1y}\right)d_{1}}\cdot 4k_{1y}k_{2y}\cdot \frac{1}{\gamma },$$

(A7) $$\begin{array}{c} g_{21}=e^{i\left(k_{1y}y-k_{2y}y^{\prime }\right)}\cdot e^{i\left(k_{2y}-k_{1y}\right)d_{1}}\cdot 2k_{2y}\cdot \left(k_{2y}+k_{3y}\right)\cdot \frac{1}{\gamma }\\ +e^{i\left(k_{1y}y+k_{2y}y^{\prime }\right)}\cdot e^{i\left(k_{2y}-k_{1y}\right)d_{1}}\cdot e^{-2ik_{2y}d_{2}}\cdot 2k_{2y}\cdot \left(k_{2y}-k_{3y}\right)\cdot \frac{1}{\gamma }, \end{array}$$

(A8) $$\begin{array}{c} g_{22}=e^{-ik_{2y}\left(y+y^{\prime }\right)}\cdot e^{2ik_{2y}d_{1}}\cdot \left(k_{2y}-k_{1y}\right)\cdot \left(k_{2y}+k_{3y}\right)\cdot \frac{1}{\gamma }\\ +e^{ik_{2y}\left(y-y^{\prime }\right)}\cdot e^{2ik_{2y}\left(d_{1}-d_{2}\right)}\cdot \left(k_{2y}-k_{1y}\right)\cdot \left(k_{2y}-k_{3y}\right)\cdot \frac{1}{\gamma }\\ +e^{ik_{2y}\left(y^{\prime }-y\right)}\cdot e^{2ik_{2y}\left(d_{1}-d_{2}\right)}\cdot \left(k_{2y}-k_{1y}\right)\cdot \left(k_{2y}-k_{3y}\right)\cdot \frac{1}{\gamma }\\ +e^{ik_{2y}\left(y+y^{\prime }\right)}\cdot e^{-2ik_{2y}d_{2}}\cdot \left(k_{2y}+k_{1y}\right)\cdot \left(k_{2y}-k_{3y}\right)\cdot \frac{1}{\gamma }, \end{array}$$

(A9) $$\begin{array}{c} g_{23}=e^{-i\left(k_{2y}y^{\prime }+k_{3y}y\right)}\cdot e^{2ik_{2y}d_{1}}\cdot e^{i\left(k_{3y}-k_{2y}\right)d_{2}}\cdot 2k_{2y}\cdot \left(k_{2y}-k_{1y}\right)\cdot \frac{1}{\gamma }\\ +e^{i\left(k_{2y}y^{\prime }-k_{3y}y\right)}\cdot e^{i\left(k_{3y}-k_{2y}\right)d_{2}}\cdot 2k_{2y}\cdot \left(k_{2y}+k_{1y}\right)\cdot \frac{1}{\gamma }, \end{array}$$

(A10) $$g_{31}=e^{i\left(k_{1y}y-k_{3y}y^{\prime }\right)}\cdot e^{i\left(k_{3y}-k_{2y}\right)d_{2}}\cdot e^{i\left(k_{2y}-k_{1y}\right)d_{1}}\cdot 4k_{2y}k_{3y}\cdot \frac{1}{\gamma },$$

(A11) $$\begin{array}{c} g_{32}=e^{-i\left(k_{2y}y+k_{3y}y^{\prime }\right)}\cdot e^{i\left(k_{3y}-k_{2y}\right)d_{2}}\cdot e^{2ik_{2y}d_{1}}\cdot 2k_{3y}\cdot \left(k_{2y}-k_{1y}\right)\cdot \frac{1}{\gamma }\\ +e^{i\left(k_{2y}y-k_{3y}y^{\prime }\right)}\cdot e^{i\left(k_{3y}-k_{2y}\right)d_{2}}\cdot 2k_{3y}\cdot \left(k_{2y}+k_{1y}\right)\cdot \frac{1}{\gamma }, \end{array}$$

(A12) $$\begin{array}{c} g_{33}=e^{-ik_{3y}\left(y+y^{\prime }\right)}\cdot e^{2i\left(k_{2y}d_{1}-k_{2y}d_{2}+k_{3y}d_{2}\right)}\cdot 4k_{2y}k_{3y}\cdot \frac{k_{2y}-k_{1y}}{k_{3y}+k_{2y}}\cdot \frac{1}{\gamma }\\ +e^{-ik_{3y}\left(y+y^{\prime }\right)}\cdot e^{2ik_{3y}d_{2}}\cdot \frac{k_{3y}-k_{2y}}{k_{3y}+k_{2y}}. \end{array}$$

The variable denoted as γ is defined for a simple representation in the expressions and it is given as follows:

(A13) $$\gamma =\left(k_{2y}+k_{1y}\right)\cdot \left(k_{2y}+k_{3y}\right)-\left(k_{2y}-k_{1y}\right)\cdot \left(k_{2y}-k_{3y}\right)\cdot e^{2ik_{2y}\left(d_{1}-d_{2}\right)}.$$
